# Temporal focus, dual-system self-control, and college students’ short-video addiction: a variable-centered and person-centered approach

**DOI:** 10.3389/fpsyg.2025.1538948

**Published:** 2025-03-24

**Authors:** Yang Liu, Yaqing Huang, Lan Wen, Peng Chen, Shuyue Zhang

**Affiliations:** ^1^Faculty of Education, Guangxi Normal University, Guilin, China; ^2^Beibu Gulf University, Qinzhou, China; ^3^Guangdong University of Foreign Studies, Guangzhou, Guangdong, China; ^4^Qinzhou Preschool Teachers College, Qinzhou, China; ^5^Guangxi College and University Key Laboratory of Cognitive Neuroscience and Applied Psychology, Guilin, China

**Keywords:** time focus, self-control, short video addiction, variable-centered, person-centered

## Abstract

**Background:**

Short video addiction has become increasingly prevalent among college students. It can negatively impact their physical and mental health, yet its influencing factors and underlying mechanisms require further exploration. Time focus and self-control are recognized as critical determinants in shaping addictive behaviors.

**Objective:**

Grounded in the I-PACE theory, this study examines the relationship between emotional and cognitive responses (various temporal focuses and dual systems of self-control) and short video addiction, while also investigating the mediating roles of inhibitory and initiation control.

**Methods:**

Methodologically, it integrates both variable-centered and person-centered approaches, utilizing the Time Focus Scale, Multidimensional Self-Control Scale, and Short Video Addiction Scale. A total of 2,239 university students participated in the survey.

**Results:**

The results revealed the following: (1) Past and present time focus were positively correlated with short video addiction, while future time focus showed a negative correlation. Inhibitory self-control was positively associated with short video addiction, whereas initiatory self-control was negatively correlated. Variable-centered analysis demonstrated that past and present time focus positively predicted short video addiction, with inhibitory self-control mediating the relationship between these time orientations and addiction. Conversely, initiatory self-control played a mediating role between future time focus and addiction risk, with a negative predictive effect on the likelihood of short video addiction. (2) Person-centered analysis identified four categories of short video addiction: non-addicted (12.68%), low-risk addiction (34.21%), moderate-risk addiction (42.20%), and high-risk addiction (10.89%). (3) Logistic regression analysis indicated that students with excessive past and present time focus were more likely to fall into the high-risk addiction category, while those employing inhibitory self-control strategies were more likely to be categorized into low, moderate, or high-risk addiction groups. Students utilizing initiatory self-control were less likely to develop high-risk addiction. Female students were more likely than male students to fall into the low, moderate, or high addiction categories, and only children were more likely to belong to the moderate or high-risk addiction categories than non-only children.

**Conclusion:**

This study emphasizes the pivotal role of time focus and dual-system self-control in the intervention and prevention of short video addiction,further highlighting the role of emotional and cognitive responses in the development of short-video addiction. The implications of the findings, as well as the limitations of the study, are also discussed.

## Introduction

According to the 53rd Statistical Report on the Development of the Internet in China (2023), the number of internet users reached 1.092 billion, representing nearly 80% of the population. The number of online video users reached 1.067 billion, while the number of short video users totaled 1.053 billion. Short video addiction has emerged as a significant concern, following the growing prevalence of internet addiction. Numerous studies have indicated that adolescents, particularly college students, are highly susceptible to excessive internet use (Davis, 2001; Zhang et al., 2023). Research has shown that the addiction and dependence rates among college students for short videos range from 21.63 to 31.99% (Li et al., 2021). Addiction to short videos can negatively impact the physical and mental health of college students, leading to depression, anxiety, insomnia, and other issues (Dong et al., 2023; Zhang et al., 2023). Furthermore, the use of short videos can distort an individual’s perception of time, reducing both learning and work efficiency (Qin et al., 2022). In comparison to traditional social media, short videos exhibit addictive properties due to their high information density and low cost of participation. The determinants of excessive short-video use and their interrelationships may differ from those associated with SNS addictions (Zhang et al., 2019). Therefore, it is crucial to gain a deeper understanding of the factors contributing to excessive short-video app use and the interrelated relationships among the key constructs.

In addition, the I-PACE model (Brand et al., 2016, 2019) serves as a specialized framework for examining irrational behaviors in human-technology interactions. This model integrates individual characteristics, emotional and cognitive responses, executive functioning, and decision-making processes, forming a robust theoretical foundation for understanding internet addiction behaviors. It emphasizes the critical role of emotional and cognitive responses alongside executive functioning. Temporal focus, as a factor of emotional and cognitive responses, influences decision-making related to executive functioning (self-control), offering valuable clinical insights into the dynamics of short-video addiction. Existing research has investigated the relationship between time and internet addiction from various perspectives, including time insight (Chavarria et al., 2015), time management tendencies (Scherr and Wang, 2021), and time distortion (Zhu et al., 2022; Yang et al., 2024), revealing a strong connection between temporal factors and Internet addictive behaviors. Whether findings from other addiction studies apply to short video addiction remains unclear, as comprehensive investigations into the role of time focus—a measure of attention distribution across past, present, and future—in short video addiction are lacking (Zimbardo and Boyd, 1999; Li, 2018; Zhu et al., 2022). As an important decision execution variable, self-control plays an important mediating role, has been found to significantly mitigate addictive behaviors (Baumeister, 2003; Mao et al., 2022; Li et al., 2024; Zhang et al., 2024). Since the introduction of dual-dimensional self-control, studies have emphasized the differing roles of inhibitory and initiatory controls in self-regulation (Baumeister, 2003; De Boer et al., 2011; Xiong and Sun, 2017; Nilsen et al., 2020; Chen et al., 2023). The I-PACE revised model also mentions that inhibitory control works only in the early stages of addictive behavior (Brand et al., 2019). Therefore, further research is necessary to determine how these dimensions impact the relationship between time focus and short-video addiction. Moreover, it dissects self-control within executive functions into two components: inhibitory control and initiatory control. This distinction allows for a detailed discussion of their respective roles in the relationship between time focus and short-video addiction. Therefore, from the perspective of different emotional responses and different executive function decisions, this study further enriched the understanding of I-PACE theory by sorting out their relationships.

### Time Focus and Short Video Addiction

Time focus is a concept used to describe the differences in temporal perspectives among individuals, reflecting the degree to which individuals allocate their attention to the past, present, and future, and represents one dimension of temporal insight (Zhu et al., 2022). Research indicates consistency in the temporal perspectives of various addictive behaviors; for example, addicts exhibit negative tendencies toward both past and present time perspectives compared to non-addicted individuals (Chittaro and Vianello, 2013; Stolarski et al., 2015) and future-oriented time perspective may help prevent and reduce such behaviors (Chavarria et al., 2015; Zhang et al., 2020). This relationship has been confirmed in internet addiction (Chittaro and Vianello, 2013) and mobile phone addiction (Jung and Han, 2014). However, there are exceptions (Kleftaras and Georgiou, 2014), such as positive past perspectives being unrelated to addictive behaviors (MacKillop et al., 2006), and inconsistencies between future focus and addictive behaviors (Kooij et al., 2018). Therefore, the relationship between different time focuses and short video addiction, as well as the underlying psychological mechanisms, requires further investigation.

The past time focus refers to an individual’s inclination to dwell on past events and experiences, particularly as reflected through emotional recollections (Zhu et al., 2022). Solomon and Corbit's (1974) opponent-process theory offers an explanation for emotional responses and addictive behaviors, using the interplay of positive and negative emotions to illustrate the underlying causes of dependency in specific systems (Turel and Serenko, 2012; Tian et al., 2023). This theory complements the I-PACE model by clarifying the role of emotions and cognition in short-video addiction. Early research indicates that individuals with a strong focus on past positive or negative experiences may experience psychological and behavioral consequences, with excessive immersion in positive memories leading to adverse outcomes (Hao et al., 2023). Additionally, studies demonstrate significant positive correlations between a negative past focus and addictive behaviors such as internet addiction (Barnett et al., 2013), and alcohol addiction (Hao et al., 2023). Short-video platforms employ specialized algorithms to deliver immersive and rapid pleasurable experiences (Zhao, 2021; Lu et al., 2022). Those who have experienced these pleasurable sensations often recall the feelings, increasing their desire for repeated engagement. Over time, as the pleasure diminishes, opposing emotional reactions such as withdrawal symptoms or cravings for pleasure emerge. Real-world psychological or external factors may temporarily interrupt short-video use; however, the removal of stimuli weakens positive reinforcement and triggers negative reinforcement, leading to emotions such as anger and stress (Tian et al., 2023). Individuals with a strong past-time focus may intensify their emotional counter-reactions, prompting them to seek relief repeatedly. Under conditions of stress, loneliness, or depression, such recollections can amplify, further increasing the desire to reengage with short videos (Ren et al., 2016; Tu et al., 2023; Qu et al., 2024). In conclusion, short video addiction stems from the interaction of positive emotions rooted in past experiences, the progressive emergence of withdrawal symptoms, and emotional responses such as anger and stress elicited by the removal of stimuli. Based on these findings, Hypothesis 1a is formulated: Past time focus is positively associated with and predictive of short-video addiction.

The present time focus describes an individual’s tendency to emphasize current events and experiences, prioritizing immediate sensations and emotions (Zhu et al., 2022). Uses and gratifications theory, a foundational framework in communication research, explains how individuals engage with media to fulfill perceived needs shaped by psychological, societal, and cultural influences (Tian et al., 2023). People with a present time focus often turn to media for immediate gratification, such as watching entertainment videos, playing games, or browsing social media. This behavior aligns with the “entertainment” and “relaxation” needs identified in the uses and gratifications model. Present-oriented individuals may also use media to escape unpleasant realities, resonating with the “escapism” and “emotional solace” needs of the theory. Research suggests that these individuals’ attitudes toward fate significantly impact their mental health and habits, predisposing them to addiction as they seek instant rewards (Ciccarelli et al., 2016). Both hedonistic and fatalistic present orientations are strongly associated with gambling, internet, and alcohol addictions (Hao et al., 2023). Short videos, characterized by their brevity, engaging content, and high entertainment value, cater effectively to the entertainment needs of present-focused individuals. The fleeting pleasure derived from watching these videos reinforces their desire to reengage, creating a self-reinforcing cycle of instant gratification. Present-hedonistic individuals often neglect long-term consequences and struggle with self-control, inadvertently spending excessive time on short videos. Additionally, short videos provide compensatory satisfaction, meeting the social and emotional needs of young users with high expectations for social interaction, thus promoting excessive use tendencies (Zhang et al., 2024). Consequently, Hypothesis 1b is formulated: Present time focus is positively associated with and predictive of short-video addiction.

Future time focus refers to an individual’s tendency to concentrate on future goals, plans, and outcomes, emphasizing foresight and long-term planning (Zhu et al., 2022). The expectancy-value theory posits that individuals’ motivated behaviors are shaped by outcome expectations and value assessments (Wigfield, 1994). This perspective impacts delayed gratification by influencing the expectations and values tied to choices, guiding individuals to prioritize the value of delayed rewards over immediate gratification. Individuals with a future time focus are more likely to consider the long-term consequences of their actions rather than focusing solely on immediate satisfaction. Early studies show that adolescents who deliberate on their future prioritize long-term outcomes and are less inclined toward risky behaviors (Kooij et al., 2018). Future-oriented individuals are better positioned to avoid addiction and, if affected, are more likely to seek help and recover successfully, with future orientation recognized as a protective factor against addiction (Cao et al., 2019; Voss et al., 2022). Regarding short-video addiction, studies reveal a negative association with future time focus, where lower future time orientation correlates with higher addiction levels (Mao et al., 2022). Moreover, individuals with a positive future orientation often exhibit a strong sense of life meaning, enabling them to better regulate their short-video usage and avoid excessive consumption (Li et al., 2024). Accumulating evidence suggests that focusing on the future may serve as a safeguard against short-video addiction. Therefore, Hypothesis 1c is formulated: Future time focus is negatively correlated with short-video addiction and predicts a reduction in such behavior.

### The mediating role of dual-system self-control

The dual-process theory posits that self-control is not merely about controlling and adjusting behavior; rather, it is a complex concept involving multiple cognitive processing stages, including inhibitory self-control and initiatory self-control. The dual-system (or dual-process) model of self-control has been corroborated by numerous studies (De Boer et al., 2011; Gillebaart and De Ridder, 2015; Chen et al., 2023).

Inhibitory self-control primarily involves the suppression of various inappropriate impulses that arise internally, and is a passive process of stopping (Dong et al., 2024). Individuals with a tendency toward a past time focus are more likely to choose inhibitory control, as past memories may trigger negative emotions. To avoid the impact of these negative emotions on current behavior, individuals may choose to suppress their emotional responses or negative thoughts. However, frequent use of inhibitory control may lead to the excessive depletion of control resources, thereby diminishing its effectiveness and reinforcing the awareness that fosters addictive behaviors (Li et al., 2021). The strength model of self-control asserts that self-control engages limited psychological resources. Excessive consumption of these resources can impair individuals’ self-control in other areas, contributing to issues like internet addiction (Nilsen et al., 2020). Participants with a present-oriented focus are more likely to prioritize immediate pleasure over future concerns, making it difficult to exercise effective control to resist undesirable behaviors. Simultaneously, a present-oriented focus is linked to increased impulsivity, which depletes inhibitory control resources, making it harder to resist current temptations and leading to greater involvements (MacKillop et al., 2006; Nigro et al., 2017). Studies on college students with mobile phone addiction have found that those with higher addiction levels exhibit poorer inhibitory control than their peers with lower addiction levels (Wang et al., 2015). Emotional and cognitive depletion among college students may impair their inhibitory control, with students suffering from mobile phone addiction being particularly vulnerable to cognitive depletion (Wu et al., 2024). In summary, Research Hypothesis 2a suggests that inhibited self-control is positively associated with short video addiction and mediates the relationship between past and present time focus and short video addiction.

In contrast, Initiative self-control involves more reflection on self-cognition, planning, and adjustment, and is an actively initiating process (Dong et al., 2024). It entails an “active self” capable of prioritizing long-term goals over short-term desires (Gillebaart and De Ridder, 2015). The Construal Level Theory (CLT) posits that the way individuals mentally represent future events influences their behavioral responses. Self-control can mitigate temptations, thereby counteracting adverse psychological reactions and interpretational biases (Trope and Liberman, 2003). High-quality self-control has been shown to predict various positive outcomes, including increased happiness (De Ridder et al., 2011) and resistance to drug addiction (Baumeister, 2018). Research suggests that initiating self-control, as a form of active self-regulation, can mitigate short video addiction, particularly among individuals with a future-oriented time perspective (Przepiorka and Blachnio, 2016; Xie and Du, 2023). In summary, Research Hypothesis 2b posits that initiatory self-control is negatively associated with short video addiction and mediates the relationship between future-oriented time perspective and short video addiction.

### The current situation of short video addiction

Recent research has examined short video addiction and its associated factors, but most studies have employed a variable-centered approach, treating short video addiction as a homogeneous phenomenon. This approach may lack precision due to the assumption of participant homogeneity, failing to account for individual heterogeneity and not fully utilizing the limitations of indicators. Latent Profile Analysis (LPA) is an individual-centered statistical method that classifies individuals into distinct subtypes based on observed indicators, facilitating the exploration of within-group diversity to enhance researchers’ understanding of subgroup characteristics (Wang and Hanges, 2011). However, the landscape of short video addiction among college students is complex (Liu et al., 2022), with distinct subtypes identifiable based on individual response patterns, including three subtypes (Pan et al., 2024) and four subtypes (Yu et al., 2022; Liu et al., 2024) and five subtypes (Li et al., 2024). Therefore, Hypothesis 3a of this study posits that college students’ short video addiction exhibits heterogeneity. Hypothesis 3b posits that different subgroups exhibit variations in time focus and self-control, offering more precise and individualized explanations for previous research. The study also examines the influence of demographic variables, such as gender and only-child status, on short video addiction subtypes. Early studies have shown that male internet gaming addiction is significantly higher than that of females (Yu et al., 2022; Tu et al., 2023). With regard to gender, several studies have indicated that short video addiction is more prevalent among female students (Yu et al., 2022). Exploring gender differences in short video addiction can provide additional validation of this issue, highlighting significant differences in internet behavior tendencies across genders. This could also draw attention from relevant authorities to the issue of female internet addiction. Additionally, internet addiction is less pronounced in only-child groups (Meng et al., 2024). Investigating the differences between only children and non-only children can further elucidate how family background and upbringing influence self-control and addiction risk related to short videos. For instance, interventions for non-only children may require a greater focus on fulfilling their social needs, rather than simply reducing short video usage time. Moreover, these findings help us recognize the need for personalized and differentiated strategies when addressing short video addiction, taking into account individual background differences. Therefore, Hypothesis 3c is proposed: Gender and only-child status are related to college students’ short video addiction subtypes.

In summary, theoretically, this study builds on the I-PACE model by integrating it with the Opponent Process Theory, the Uses and Gratifications Theory, and the Expectancy-Value Theory, further highlighting the role of emotional and cognitive responses in the development of short-video addiction. From a content perspective, the research provides a fresh lens for comprehensively analyzing the connections between affective and cognitive responses (past, present, and future time focus) and short-video addiction. Additionally, it categorizes self-control within executive functions into inhibitory and initiating controls, thoroughly discussing their varying roles in linking time focus and short-video addiction. This enriches and expands the I-PACE framework. From a methodological standpoint, this research combines variable-centered and person-centered techniques. This approach mitigates the risk of disregarding individual variability and differences, which might otherwise obscure significant patterns of short-video addiction within subgroups. By integrating these methods, ultimately providing valuable empirical support for interventions targeting short video addiction among college students.

### The current study

**Figure 1:**
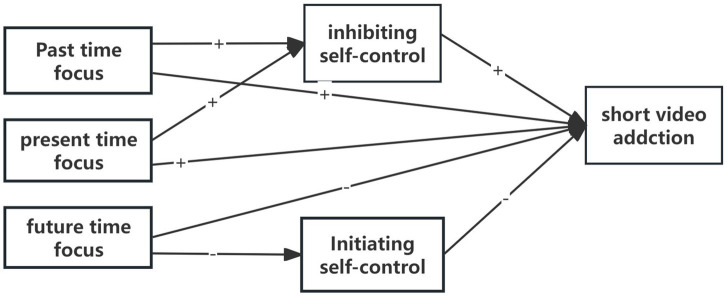
The proposed serial mediation model among variables Note. “+” and “-” represent positive and negative associations between variables, respectively.

The primary objective of this study is to examine the relationship between various time focuses, multidimensional self-control, and short video addiction. To achieve this, a comprehensive analytical approach was employed, focusing on variable-centered analysis to examine the potential mediating role of multidimensional self-control in the relationship between three distinct time focuses and short video addiction. The research design is presented in Figure 1. Furthermore, latent profile analysis (LPA) was adopted as an individual-centered approach to further elucidate the heterogeneity in short video addiction among college students and identify key influencing variables across various subgroups. Following the LPA, multinomial logistic regression was applied to assess the associations between gender, only-child status, past time focus, present time focus, future time focus, inhibitory self-control, initiating self-control, and short video addiction.

## Methods

### Participants

A questionnaire survey was administered to college students from eight universities in April 2024. A convenience sampling technique was employed for participant recruitment. A total of 2,500 first-year and second-year university students were recruited through the distribution of recruitment information via peer networks. All participants voluntarily signed an informed consent form after comprehensively understanding the study’s purpose and procedures. After excluding participants who provided patterned responses (i.e., selecting the same option for more than 80% of the questions) and those whose completion time was too short (defined as less than 2 min), 2,239 questionnaires were considered valid, resulting in a valid response rate of 89.56%. G*Power was used to estimate the sample size and calculate the minimum required sample size. Based on preliminary research, an alpha level of 0.05, a power of 0.80, and an effect size of 0.04 were set. The minimum required sample size was determined to be 190. The final sample size of 2,239 significantly exceeds this threshold, ensuring the reliability of the study’s results. The sample was drawn from various regions, including Guangxi, Guangzhou, Wuhan, and Yunnan, enhancing its representativeness. The sample included 948 males and 1,291 females, with an average age of 19.16 years (SD = 0.81). Data were collected through the Questionnaire Star platform. The survey was administered online in a centralized group setting during class meetings, with the assistance of counselors, on a class-by-class basis. Prior to the survey, detailed instructions were provided to participants, emphasizing that the results would be strictly confidential and used solely for scientific research purposes. Participants were informed that they could withdraw from the study at any point. The questionnaire took approximately 5 min to complete. This study received approval from the Academic Ethics Committee of the College of Educational Sciences at Guangxi Normal University (approval number: 2024–001).

### Measures

#### The Short Video Addiction Scale

This research used the Short Video Addiction Scale, adapted from Leung’s Mobile Phone Addiction Index (MPAI) (Qin et al., 2019), which includes 14 items across four dimensions: withdrawal (5 items, e.g., “You feel uneasy without short videos”), escapism (3 items, e.g., “You watch short videos when you feel lonely”), loss of control (4 items, e.g., “You fail to reduce time spent on short videos”), and inefficiency (2 items, e.g., “Short videos lower your productivity”). The scale is scored on a 1–5 Likert scale, with the total score ranging from 14 to 70, where higher scores indicate greater addiction severity. In the present study, the *Cronbach’ s α* were 0.861, 0.828, 0.761, and 0.872 for each dimension, respectively.

#### The Temporal Focus Scale (TFS)

This study utilized the Temporal Focus Scale, developed by Shipp et al. (2009) and revised by Zhu et al. (2022). The scale consists of 12 items, divided into three dimensions: four items for past focus (e.g., “I tend to recall past experiences”), four items for present focus (e.g., “I focus on the present moment”), and four items for future focus (e.g., “I pay attention to what will happen in the future”). The scale employs a 5-point Likert scale (1 = “Never,” 2 = “Rarely,” 3 = “Sometimes,” 4 = “Often,” 5 = “Always”), with higher scores indicating greater attention to a specific time period. In the present study, the *Cronbach’ s α* were 0.934, 0.907, and 0.911, respectively.

#### The Brief Multidimensional Self-Control Scale (BMSCS)

The Brief Multidimensional Self-Control Scale, revised by Dong et al. (2024) from Tangney’s original MSCS, consists of 8 items across two dimensions: inhibitory self-control (e.g., “I struggle to begin tasks”) and initiating self-control (e.g., “I understand what’s needed to achieve my goals”). The scale is rated on a 5-point Likert scale, where higher scores reflect greater self-control. In the present study, the *Cronbach’ s α* were 0.853 and 0.834, respectively.

### Statistical analyses

Meghan K. offered a tutorial on calculating univariate and multivariate skewness and kurtosis using a newly developed web application (Cain et al., 2017). The results revealed skewness (*b = 3.34, z = 1245.19, p < 0.05*) and kurtosis (*b = 23.14, z = 35.17, p < 0.05*), indicating a deviation from multivariate normality. To validate the research hypotheses, the study aimed to examine the psychological and behavioral factors influencing short video addiction through a four-step analysis. First, descriptive statistics and correlation analyses were performed. Second, Structural Equation Modeling (SEM) using AMOS 24.0 was employed to examine the relationships between temporal focus, self-control systems, and short video addiction.

This method assesses the mediation effect by resampling with replacement from the original dataset while maintaining the same sample size, thereby improving statistical efficiency. In this study, 5,000 random samples were generated to calculate the 95% confidence interval (CI) for the mediation effect. The indirect effect was considered statistically significant if the 95% CI did not include zero. To assess model fit, the following indices were examined: X^2^/df (recommended range: 2–5), *p*-value, root mean square error of approximation (RMSEA; < 0.06), standardized root mean square residual (SRMR; < 0.08), goodness of fit index (GFI; > 0.90), comparative fit index (CFI; > 0.95), and Tucker–Lewis Index (TLI; > 0.95). Third, Latent Profile Analysis (LPA) with Mplus 8.3 was employed to identify patterns of short video addiction among college students. The model selection criteria were based on the following indices: AIC, BIC, and ABIC, which favor lower values; IMR and BLRT, which show significant *p*-values (< 0.05), indicating that adding a profile improves model fit; and entropy values closer to 1, which typically signify more effective classification. Finally, cross-logical regression analysis with SPSS 21.0 was conducted to assess the impact of temporal focus, self-control, gender, only-child status, and addiction characteristics. These findings contribute to the understanding of the psychological and behavioral factors underlying short video addiction and provide a theoretical basis for future interventions.

## Results

### Preliminary analyses

Harman’s single-factor test was used to examine the presence of significant common method bias effects. A factor analysis was conducted on all items, resulting in seven factors with eigenvalues greater than 1. The first factor accounted for 18.14% of the total variance, which is below the critical threshold of 40% (Zhou and Long, 2004), indicating no significant common method bias issue. Descriptive statistics and correlations of the variables are reported in Table 1, showing that past and present time focus are positively correlated with short video addiction (*r = 0.13, p < 0.01; r = 0.11, p < 0.01*), while future time focus is negatively correlated with short video addiction (r = −0.08, *p* < 0.05). Inhibitory control is significantly positively correlated with short video addiction (*r = 0.25, p < 0.01*), whereas initiation control is negatively correlated with short video addiction (*r = −0.64, p < 0.01*). Past (*r = 0.25, p < 0.01*), present (*r = 0.16, p < 0.01*), and future (*r = 0.16, p < 0.01*) time focus are positively correlated with inhibitory control; they are also positively correlated with initiation control (*r = 0.64, p < 0.01; r = 0.69, p < 0.01; r = 0.70, p < 0.01*).

**Table 1:** Descriptive statistics and correlation matrix (*n* = 2239).

	*M*	*SD*	1	2	3	4	5	6
1 Past time focus	15.48	3.05	1					
2 Present time focus	15.06	2.99	0.84**	1				
3 Future time focus	14.76	3.03	0.82**	0.84**	1			
4 Inhibitory control	12.048	3.37	0.25**	0.16**	0.16**	1		
5 Initiation control	13.91	2.89	0.64**	0.69**	0.70**	0.12**	1	
6 Short video addiction	35.49	10.78	0.13**	0.11**	−0.08*	0.37**	−0.09**	1

^*^P < 0.05, **P < 0.01.

### Mediation analyses: variable-centered approach

**Figure 2:**
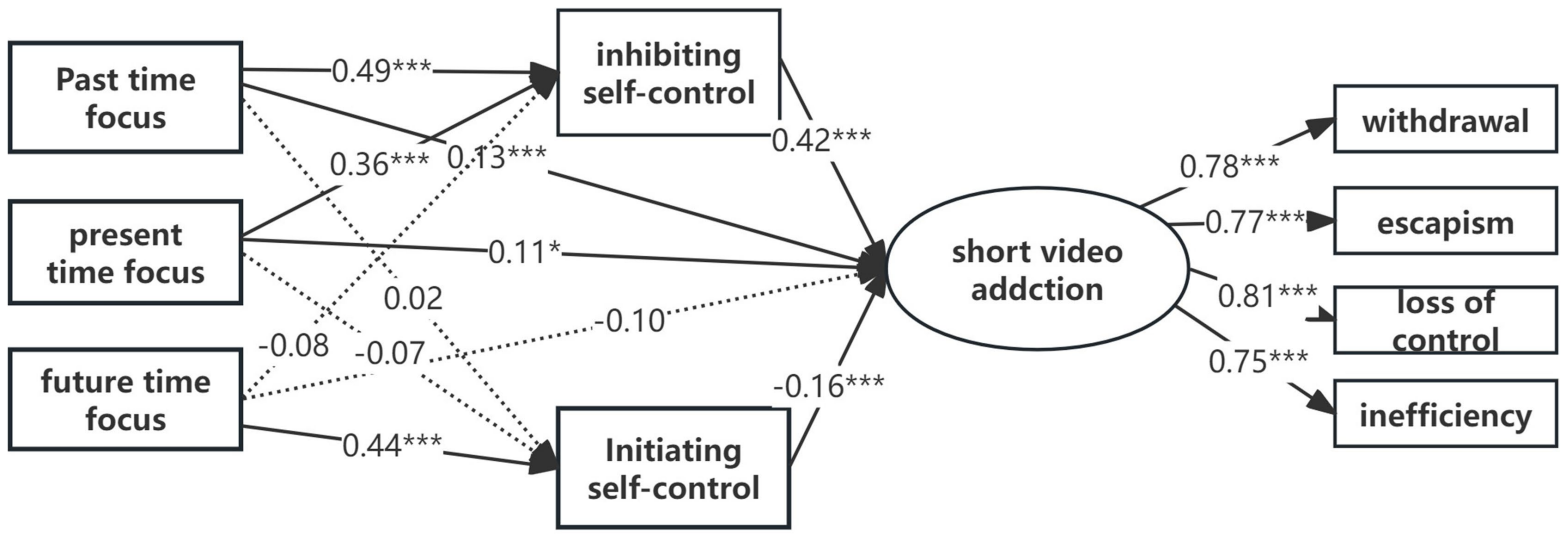
Relationships between different time focus, inhibiting self-control, Initiating self-control, and short video addiction. The dotted line represents non-significant relationship. ***p < 0.001, *p < 0.05.

A parallel mediation model was constructed using AMOS 24.0 through structural equation modeling, with past focus, present focus, and future focus as independent variables, inhibitory control and initiation control as mediating variables, and short video addiction as the outcome variable. Observed indicators included loss of control, withdrawal, escapism, and inefficiency. The results are presented in Figure 2. The model fits well, with model fit indices as follows: χ^2^/df = 10.47, CFI = 0.96, TLI = 0.95, RMSEA = 0.067, 90% CI = [0.063, 0.071], and SRMR = 0.048.With respect to the relationship between time focus, self-control, and short video addiction, a significant positive association between past time focus and short video addiction (*β = 0.13, SE = 0.05, p < 0.001*) was observed, H1a was supported in this study; a significant positive association between present time focus and short video addiction (*β = 0.11, SE = 0.05, p < 0.001*) was observed, H1b was supported in this study; but no significant association was found between future time focus and short video addiction (*β = −0.01, SE = 0.05, p = 0.834*) was observed, H1c was not supported in this study.

Further mediation analysis was performed using AMOS 24.0, employing the bias-corrected percentile bootstrap method (Fang et al., 2012), which estimate the 95% confidence interval (CI) for the mediation effect, testing its statistical significance. The results indicate that inhibitory control mediates the relationship between past focus and short video addiction [*indirect effect = 0.18, 95% confidence interval (CI) = (0.14, 0.23), effect sizes = 66.22%*], as well as the relationship between present focus and short video addiction [*indirect effect = 0.08, 95% confidence interval (CI) = (0.04, 0.13), effect sizes = 57.89%*]. Initiation control mediates the relationship between future focus and short video addiction [*indirect effect = −0.06, 95% confidence interval (CI) = (−0.11, −0.02), effect sizes = 41.31%*]. The findings suggest that inhibitory control mediates the impact of past and present focus on short video addiction, while initiation control mediates the impact of future time focus on short video addiction.

### Latent profile analyses

Latent Profile Analysis (LPA) is used to aid researchers in identifying the specific characteristics of each subtype. The results demonstrate that, beginning with a one-class model and progressively increasing the number of profiles from 1 to 6, the fit indices for each model are presented in Table 2. This process involves comparing the AIC, BIC, and aBIC values of various models, along with evaluating the classification accuracy (Entropy value). While the three-class model performed well on certain indicators, it was somewhat insufficient in explaining the complexity of the data (Rindskopf, 2003). Information criteria (AIC, BIC, ABIC): The smaller the value, the better. Moreover, the significant values of the LMR and BLRT indices indicate that the four-class model outperforms the three-class model. The four-class model offers a significant advantage in explaining the data, where each class can be assigned a specific interpretation. It allows for more accurate identification of different categories of short video addiction users, such as mild, moderate, severe addiction, and non-addicted users. An examination of the diagonal indices in Table 3 revealed that the predictive model was reliable, with positive predictive values ranging from 92 to 97%.

**Table 2:** Model fit indices of the of college students’ short video addiction.

Model	*AIC*	*BIC*	*aBIC*	*Entrop*	*LMR*	*BLRT*	Percentage of sample
1-class	92705.423	92865.408	92776.448	-	-	-	-
2-class	82382.333	82828.026	82691.408	0.911	<0.01	<0.01	0.42/0.58
3-class	79448.160	79779.559	79595.284	0.904	<0.01	<0.01	0.14/0.45/0.40
4-class	77577.937	77995.043	77763.111	0.904	<0.01	<0.01	0.13/0.42/0.34/0.11
5-class	76583.588	77086.401	76806.811	0.885	0.04	0.04	0.33/0.13/0.22/0.23/0.09
6-class	75777.266	76365.786	76308.538	0.875	0.57	0.58	0.13/0.25/0.15/0.21/0.09/0.18

**Table 3:** Most likely latent profile membership (row) by latent profile (column).

Profile	*N*	Percentage	Attribution probability			
			C1	C2	C3	C4
C1	285	12.68%	0.970	0.030	0.000	0.000
C2	766	34.21%	0.011	0.940	0.049	0.000
C3	945	42.20%	0.000	0.038	0.942	0.020
C4	244	10.89%	0.000	0.000	0.074	0.926

**Figure 3:**
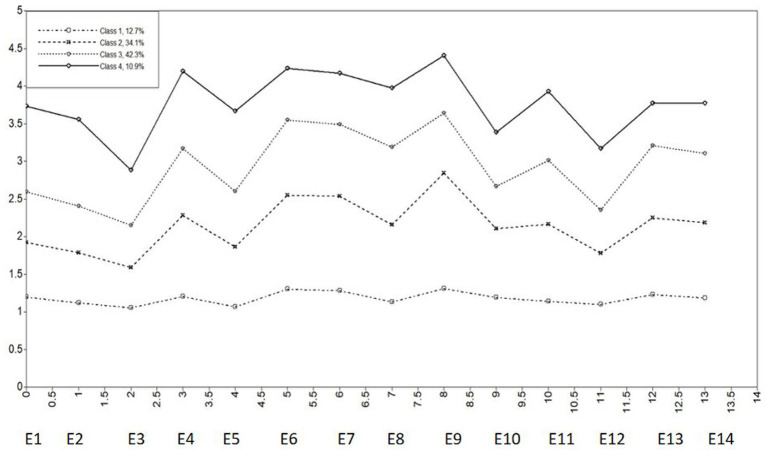
Latent profile analysis of short video addiction: Four-profile solution. E1–E14 = items 1–14 of the short video addiction scale.

Internet addiction profiles were derived from the patterns in the means of the Internet addiction variables. The means of the indicator variables for each profile are presented in Figure 3. Profile 1, comprising 12.68% of the sample (*n = 285*), was characterized by low levels across all indicators. This profile was labeled as the Non-Addiction Profile. Profile 2, comprising 34.21% of the sample (*n = 766*), was labeled as the Low-Risk Addiction Profile due to its low mean scores across all 14 Internet addiction items. Profile 3, comprising approximately 42.2% of the sample (*n = 945*), included individuals with a moderate probability of Internet addiction and was labeled as the Medium-Risk Addiction Profile. Profile 4, comprising approximately 10.89% of the sample (*n = 244*), was characterized by the highest mean scores on the Internet addiction items and was labeled as the High-Risk Addiction Profile.

To assess the validity of the profile classification, this research examined its association with the dimensions of achievement goal orientations using analyses of covariance. Table 4 presents the means and standard deviations (SD) for each goal orientation. Using the four dimensions of short video addiction as the dependent variables and the four latent categories as the independent variables in a one-way ANOVA, the results indicate that: the main effect of the latent categories is significant for all four dimensions. The differences in total scores and scores on each dimension among the four latent categories of college students’ short video addiction were statistically significant (*p < 0.05*). Further pairwise comparisons indicated that C1 was lower than C2 (*p < 0.05*), C2 was lower than C3 (*p < 0.05*), and C3 was lower than C4 (*p < 0.05*).

**Table 4:** Comparison of scores in short-form video addiction and various dimensions among different types of college students (x ± s).

Profile	Withdrawal	Escapism	Loss of control	Inefficiency
C1	5.61 ± 1.19	3.67 ± 1.33	4.72 ± 1.32	2.40 ± 0.91
C2	9.42 ± 2.16	7.24 ± 1.88	8.86 ± 1.67	4.40 ± 1.50
C3	12.92 ± 2.29	10.24 ± 1.73	11.69 ± 1.78	6.33 ± 1.53
C4	18.11 ± 2.51	12.36 ± 1.63	14.93 ± 2.15	7.55 ± 1.66
F (3, 2293)	1823.622***	1588.91***	1926.88***	803.86***
LSD	1 < 2 < 3 < 4	1 < 2 < 3 < 4	1 < 2 < 3 < 4	1 < 2 < 3 < 4

***P < 0.001.

### Relationships between demographic variables, different time perspectives, self-control, and short video addiction

Multinomial logistic regression analyses were conducted with the Non-Addiction Profile as the reference group because it was the profile that included the individuals with the lowest probability (almost no) of Internet addiction. As Table 5 indicates, compared to boys, there are significant gender differences in low, medium, and high-risk addiction, with the probability for females being significantly higher than for males. Among only children, the probability of medium and high-risk addiction is higher. The results from different time perspectives show that college students with a past and present time focus have a higher probability of high-risk addiction; the self-control results show that college students using inhibitory control strategies have a higher probability of short video addiction across low, medium, and high risk levels, while those using initiatory control strategies can effectively reduce the probability of high-risk short video addiction.

**Table 5:** Results of the multinomial logistic regression predicting latent profile membership.

	C2 (Low risk)		C3 (Medium risk)		C4 (High risk)	
	OR	CI	OR	CI	OR	CI
Gender (boy)	−1.19***	0.23 ~ 0.41	−0.66**	0.39 ~ 0.69	−1.79**	0.11 ~ 0.25
Only child (yes)	0.24	0.55 ~ 1.13	0.43*	0.46 ~ 0.93	0.62*	0.45 ~ 0.93
Past time focus	0.06	0.97 ~ 1.19	0.02	0.92 ~ 1.13	0.19**	1.07 ~ 1.38
Present time focus	0.08	0.84 ~ 1.04	0.01	0.91 ~ 1.11	0.13**	1.03 ~ 1.79
Future time focus	−0.03	0.88 ~ 1.07	0.01	0.91 ~ 1.12	−0.45	0.84 ~ 1.08
Inhibitory control	0.27***	1.25 ~ 1.38	0.14**	1.11 ~ 1.21	0.44**	1.45 ~ 1.66
Initiation control	−0.04	0.89 ~ 1.03	0.01	0.94 ~ 1.08	−0.08*	1.00 ~ 1.35

OR, odds ratio, CI, confidence interval. *P < 0.05, **P < 0.01, ***P < 0.001.

## Discussion

### Relationship between time focus and short video addiction

This study examined the relationship between time focus and short video addiction, with results partially supporting the hypotheses. Specifically, past time focus positively predicted short video addiction (Hypothesis 1a), and present time focus also positively predicted short video addiction (Hypothesis 1b). Existing research indicates that both negative past focus and present time focus influence internet addiction (Chittaro and Vianello, 2013) and mobile phone addiction (Jung and Han, 2014).

The findings further confirm that past and present time focus are risk indicators for addiction (Hao et al., 2023). The results suggest that excessive focus on the past and present may contribute to short video addiction. First, past-oriented attention may lead individuals to bring their emotional experiences from the past into their present lives, thus engaging in behaviors related to those past experiences (Cao et al., 2019). The content offered by short videos is closely related to an individual’s experiences, reinforcing these experiences and increasing the risk of addiction. Secondly, individuals who focus on the past tend to derive their personal identity through social comparison, and the high social interactivity of short video platforms amplifies this social comparison effect (Chou and Edge, 2012; Hao et al., 2023). Individuals can obtain self-affirmation by watching others’ content, which in turn increases the frequency of short video consumption. Compared to other types of internet addiction (such as online gaming or social media addiction), short videos provide a more direct “emotional shortcut,” catering to personal nostalgic emotions or serving as an escape from negative feelings. Thus, an excessive focus on the past could be a risk factor for short video addiction. Conversely, individuals who focus on the present are more likely to seek instant gratification and display higher levels of impulsivity (Cao et al., 2019). This also helps explain why individuals who are more susceptible to addiction are more likely to seek immediate rewards while neglecting long-term negative consequences, thus becoming more addicted to the immediate gratification they receive (MacKillop et al., 2006; Nigro et al., 2017). Short videos offer immediate emotional rewards (e.g., humor, surprise, or relaxation), aiding users in reducing stress or anxiety (Jiang et al., 2024). Overall, attention focused on the past and present largely leads to addiction due to emotional avoidance and self-regulation issues. The I-PACE theory shows that emotional cognitive response plays an important role in short video addiction.

The relationship between future time focus and short video addiction was negative, but it did not reach statistical significance, and thus does not support H1C. The relevant research results have also been confirmed future temporal focus involves a general orientation toward the future but does not necessarily involve the concrete specification of future goals to the extent required by the self-control process (Li et al., 2024). Future time focus cannot play a protective role because it has a significant negative impact on planned desires in a state of ego depletion (Sjåstad and Baumeister, 2018). The incentive sensitization theory also mentions that due to the limited nature of attentional resources, when more attention is allocated to social information, the attention allocated to future goal information decreases (Liu et al., 2021). This reduction in attention allocation can lead people to abandon long-term goals because their current good feelings can easily override future goals. Even if individuals focus on the future at a specific moment, this focus may be temporary and does not necessarily translate into sustained behavioral change (Glasman and Albarracín, 2006). In other words, merely focusing on the future is not enough to form sufficient motivation to resist immediate gratification (Ciccarelli et al., 2016), such as the pleasure brought by short videos, even when individuals exhibit a future-oriented focus, the absence of clear, specific goals can hinder the effective application of self-control in resolving goal conflicts.

The individual-centered analysis indicates that individuals with a past or present time orientation are at an elevated risk of developing high-risk short video addiction, thus aligning with Research Hypothesis 3b. The association between past and present time orientation and high-risk short video addiction may be attributed to their connection with emotional escapism (Jiang et al., 2021), insufficient self-regulation (Chen et al., 2023), and a preference for immediate gratification. Conversely, individuals in the low- and medium-risk addiction categories demonstrate a greater capacity to manage their addiction risks, likely due to enhanced self-regulation and self-control mechanisms. These findings emphasize the pivotal role of mediating and moderating variables in shaping individual susceptibilities. Further analysis of the relationship between future time focus and short video addiction reveals that initiation control plays a crucial role in mediating this relationship. Therefore, even if an individual demonstrates a future-oriented focus, the absence of clear plans and future-oriented strategies may hinder the effective use of self-control to resolve goal conflicts. A notable finding is that past time orientation, as a predictor of short video addiction, differentiates it from other forms of internet addiction, highlighting its unique predictive value.

### The mediating role of self-control

Variable-centered research indicates that inhibitory control mediates the relationship between past and present time focus and short video addiction, aligning with Hypothesis 2a. In other words, when exerting self-control, if an individual’s thoughts are dominated by inappropriate impulses, this constitutes a passive inhibition process. However, this passive inhibition may lead to emotional restlessness, discomfort, and even regret. To regulate their emotions, individuals may continue watching short videos, gradually becoming more addicted and unable to extricate themselves. The strength model of self-control (Muraven and Baumeister, 2000) further posits that self-control relies on limited internal resources or energy. Individuals who excessively focus on the past and present, particularly those who overuse smartphones, experience greater conflict during the initial stages of inhibitory processing (Chen et al., 2023). Inhibitory control is regarded as a cognitive factor closely linked to smartphone use; an individual’s craving for specific stimuli may impair stimulus inhibition, leading to problematic behaviors (Wegmann and Brand, 2021). The effort to exert inhibitory control rapidly depletes these limited internal resources, placing individuals in a state of “ego depletion.” In a depleted state, individuals find it more difficult to inhibit impulses, and self-control is more likely to fail (Baumeister, 2003; Tan et al., 2012). The failure of inhibitory control can perpetuate this cycle, not only failing to curb the frequency of short video use effectively, but also exacerbating it. Therefore, particular attention must be given to prevent the paradox where greater inhibition leads to increased addiction.

Research suggests that proactive control mediates the relationship between future time focus and short video addiction, aligning with our hypothesis 2b. Proactive control negatively affects short video addiction, indicating a protective role in addiction. The results further suggest that, in addressing short video addiction, individuals not only attempt to inhibit impulses but also proactively implement various self-control strategies to achieve effective self-regulation (Chen et al., 2023). Success is more likely when individuals proactively implement self-control strategies, change their perceptions of temptations (Fujita and Han, 2009), and make pre-commitments before encountering temptations (Ladouceur et al., 2012). For short video addiction, preemptively setting up distractions, such as distraction and goal-reminder strategies, is particularly effective in resisting leisure cravings (Chen et al., 2023). Related research suggests that individuals with high self-control frequently implement proactive strategies aligned with long-term goals (Galla and Duckworth, 2015; Gillebaart and De Ridder, 2015). It is evident that while future time focus can help inhibit short video addiction, specific actions are necessary. Merely having future goals is insufficient to combat addiction. Therefore, for individuals with a future time focus, the importance of proactive control strategies is paramount. Emphasizing proactive control in preventing short video addiction offers valuable insights for intervention strategies.

The individual-centered analysis further supports and validates Research Hypothesis 3b. The results indicate that, compared to university students without short video addiction, individuals using inhibitory control are more likely to exhibit low, moderate, or high levels of short video addiction risk. This further suggests that the adoption of inhibitory control strategies may increase the likelihood of addiction. This occurs because inhibitory control leads to excessive self-depletion, and under cognitive depletion, individuals are more prone to impulsive decision-making (Dong et al., 2024), diminished integrity, and may even engage in unethical behaviors (Chen et al., 2023). Additionally, university students with high smartphone dependence demonstrate poorer inhibitory control compared to those with low smartphone dependence (Wang et al., 2015). Therefore, the individual-centered analysis results further underscore the significant role of inhibitory self-control in the development of short video addiction.

### Short video addiction profiles

This research explored the latent profiles of short video addiction by conducting Latent Profile Analysis (LPA). Consistent with Hypothesis 3a and previous studies, this study identified distinct patterns of short video addiction among Chinese university students. A four-profile model was developed in this study: Non-Internet Addiction Profile, Low-Risk Profile, Moderate-Risk Profile, and High-Risk Profile. This further confirms the heterogeneity of short video addiction among college students, with key influencing factors that can be analyzed across different subtypes.

### The relationship between gender, only-child status, and college students’ short video addiction

Multivariate logistic regression analysis indicates that females exhibit higher rates of low-risk, medium-risk, and high-risk short video addiction, thereby supporting research hypothesis 3C, which is further corroborated by related research (Pan et al., 2024). The reason is that short videos, compared to other forms of internet addiction, have characteristics of entertainment, social interaction, and relaxation, and females have a greater need for social interaction and entertainment compared to males (Bi et al., 2005); females have lower resilience and are more inclined to choose online forms to address personal loneliness, thus being more prone to addiction (Zhao et al., 2024). Additionally, short videos integrate self-presentation functions, such as photo-taking and beautification, which are more likely to cater to the needs of females (Wang and Wang, 2023). This result also indicates that the issue of internet addiction among females urgently needs societal attention, especially the problem of short video addiction. Compared to non-only children, only children exhibit higher rates of medium-risk and high-risk short video addiction, thereby supporting research hypothesis 3C. Only children, due to increased feelings of loneliness, are more vulnerable to short video addiction. Lacking siblings, only children may have smaller social networks compared to non-only children, which could result in greater loneliness during social interactions. This loneliness may prompt them to seek alternative social channels, such as short video platforms, to fulfill their social needs (Meng et al., 2024). Only children may develop self-centered behavioral patterns due to the exclusive enjoyment of family resources, which may affect their social interaction skills, making it difficult for them to establish and maintain interpersonal relationships in real life, thus making them more prone to become addicted to virtual social environments like short video platforms (Liu, 1999).

## Conclusion

This study combines variable-centered and person-centered approaches to investigate the impact of college students’ time focus on short video addiction and explore the mediating role of dual-system self-control. The results indicate:(1) Variable-centered analysis reveals that past and present time orientations positively predict short video addiction, with inhibitory self-control mediating the relationship between these time orientations and addiction. In contrast, proactive self-control mediates the relationship between future time orientation and addiction risk, negatively predicting the likelihood of short video addiction. (2) Person-centered analysis identifies four categories of short video addiction: non-addicted, low-risk addiction, medium-risk addiction, and high-risk addiction. (3) Logistic regression analysis reveals that students who excessively focus on past and present orientations are more likely to develop high-risk addiction, while those employing inhibitory self-control strategies are more likely to be classified into low-, medium-, and high-risk addiction categories. Students who exhibit strong proactive self-control are less likely to develop high-risk addiction. (4) Female college students are at a higher risk of low-, medium-, and high-level addiction compared to their male counterparts. Only children are more likely than non-only children to fall into medium- and high-risk addiction categories. (5) The findings of this study have theoretical significance for future research and offer practical implications for interventions targeting short video addiction. From a theoretical perspective, grounded in the I-PACE theory, this study offers a comprehensive analysis of short video addiction, deepening our understanding of its underlying mechanisms. Furthermore, it contributes to the enrichment of both the I-PACE model and Construal Level Theory, expanding their applicability in the context of digital media consumption. It underscores the distinct roles of cognitive-emotional responses and executive functions in addiction. Additionally, it emphasizes the influence of decision-making styles, inhibitory control, and initiation control on varying addiction outcomes. Moreover, it explores how emotional and cognitive responses to varying temporal focuses regulate decision-making processes prior to execution. Specifically, it highlights strategies aligned with future goals to more effectively prevent addictive behaviors (Hao et al., 2023). In the intervention process, it is essential to focus not only on guiding groups with a past and present focus but also on reducing negative recollections of the past and dependence on current impulses. On one hand, individuals will undergo training to identify and resist temptations, improving their control over impulsive behaviors. On the other hand, they will be guided to set effective and reasonable future goals. Additionally, clear and future-oriented plans and measures need to be established to enhance initiation control, which is crucial in effectively preventing short video addiction.

However, several limitations should be acknowledged. Given the limitations of the sample, the study included only first-year and second-year participants. Future research should focus on expanding the participant pool and investigating how short video addiction may involve varying influencing factors across different age groups. The study design failed to fully include the effects of personality traits (e.g., neuroticism, agreeableness, conscientiousness) and other psychological factors (e.g., depression, anxiety) on short video addiction. Future studies, incorporating the I-PACE model, can integrate personality variables alongside emotional cognitive responses and executive functions, providing a more comprehensive explanation of the internal mechanisms of short video addiction. Secondly exploring how time focus influences the long-term development of short video usage remains an important avenue for future research. Previous research suggests that a balanced temporal perspective, which integrates both past (tradition) and present orientations (Callizo-Romero et al., 2020), can enhance mental health and prevent internet addiction (Li and Lv, 2024). Future research could examine the relationship between this balanced temporal perspective and short video addiction.

## Data Availability

The raw data supporting the conclusions of this article will be made available by the authors, without undue reservation.
